# Supplementary material for: Eutrophication will increase methane emissions from lakes and impoundments during the 21st century
Source: Nat Commun. 2019 Mar 26;10:1375. doi: 10.1038/s41467-019-09100-5 (PMC6435651; doi:10.1038/s41467-019-09100-5)
Supplement: Supplementary file 1 — Supplementary Information [file 41467_2019_9100_MOESM1_ESM.pdf]

Supplementary Information

Eutrophication will increase methane emissions from lakes and impoundments during the 21st century

Beaulieu et al.

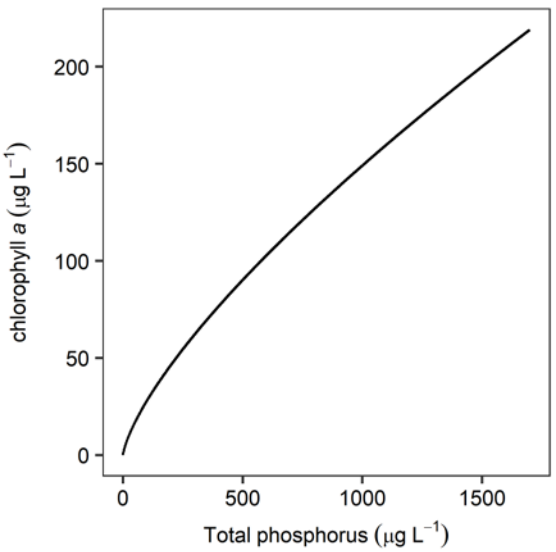

Supplementary Figure 1. **Relationship between chlorophyll *a* and total phosphorus in lakes and impoundments.** Black line represents the empirical relationship between chlorophyll *a* (chla) and total phosphorus (TP) concentration in lakes and impoundments based on measurement in 1058 systems [1]. The model is: log10(chla) = (0.7246777624 * log10(TP)) - 0.1313111819. Model predictions were transformed to linear space in this figure.

Supplementary Table 1. Details of phosphorus (P), nitrogen (N), and augmentation factors presented in Table 1 and used to parameterize the future scenarios simulated in this study. The principal mechanism of increasing eutrophication will be increases in P release from population-driven agriculture and sewage release. These increases will be further augmented by the increasing spatial extent of lakes and impoundments, increased nutrients due to intense storm- and flood-driven runoff, and the warming of water bodies due to climate change. Export or loading of N and P are assumed to be near-linearly related to concentrations in inland waters because, unless future water loads drastically increase in regions producing agricultural crops[2], loading of N and P are known to be tightly coupled with concentrations in lakes[3]. Further, concentrations of N and P have been shown to be quite tightly correlated with each other [4]. This analysis does not account for increased nutrient concentrations in warm regions due to concentration from enhanced evaporative losses[2, 5] so overall factors are likely underestimates. If a range of values was reported by the publication, the median or average is reported here and in Table 1.

Citation	Change to 2050		Change to 2100		
	N	P	N	P	Estimator and notes
**Changes in fertilizer production and nutrient runoff**					
Caraco & Cole (1999), Cole et al. (1993) combined with population growth from Samir & Lutz (2017)	1.23×	--	1.41x	--	Predictions of increased N export to oceans derived from a nitrate export model for river basins. Concentration increases in inland waters will be higher than this because these estimates are for effluent from inland waters after nutrients have moved downstream from lakes to river tributaries to the sea. Export is considered proportional to concentration because the predictions are based entirely on human population increases driving releases of nutrients from sewage and agriculture.
Bouwman et al (2013)	1.19x	1.50x	--	--	Increased run-off P from global food animal production, regardless of animal production scenarios. Manure and urine from animal production is highly mobile, containing high concentrations of N and P. Animal production is projected to be strongly correlated with export, loading, and concentrations in receiving waters.
Tilman et al. (2001)	2.70×	2.44x	--	--	Prediction of N and P-driven eutrophication increase due to expanded agriculture and population growth. These are direct estimates of increased nutrient concentrations in freshwaters based on conversion of natural lands to agriculture, losses of nutrients from fertilized crop, and untreated manure production.
Cordell et al (2009)	--	1.97×	--	3.19×	Based on the “peak P” concept concerning depleting reserves and

					the idea that 55% of all mined P is released to water. Both estimates are derived from cumulative P mined since 1950 and assume no major improvement in conservation of P and no increase in identified P rock reserves. Because most changes in nutrient concentrations in freshwaters are linked to increased loading, except in regions where water discharge is likely to increased faster than nutrient export, nutrient concentrations in global waters will climb proportionately with increased P depletion.
Samir & Lutz (2017)	1.37×	--	1.50×	--	Projected population increase without utopian assumptions, assuming a wealthy and fragmented world (SSP3). Assumes proportionality of water quality impact and population growth and no change in per capita food demands and sewage production. Concentrations are projected to rise because exports will increase. This will be true unless water discharges increase markedly in population centers and agricultural areas supporting them.

Supplementary Table 1. ***continued***

Citation<br>(*see main references*)	Change to 2050	Change to 2100	Estimator and notes
**Changes that will augment effect of changes in fertilizer production and nutrient runoff**			
Jeppesen et al (2009),<br>Sinha et al (2017),<br>Downing (2014)	1.10×	1.14×	Jeppesen et al (2009) predict a 1.09x increase in stream delivery of P due to climate change by 2100. The authors note that this is likely an underestimate of increased eutrophication because waters will be warmer and nutrient mobilization from sediments will be greater. Sinha et al. (2017) predict a 1.19× increase of N delivery to rivers and streams in the United States due to climate change and increased storms by 2100. We use the mean value from these two studies, 1.14, to represent the effect of climate change on nutrient delivery to inland waters by 2100. Downing et al. (2014) predict that by 2050 climate change will increase nutrient loading to inland waters by 1.10×. These projections are based on the fact that intense storms liberate more nutrients than the amount mobilized by the same precipitation delivered gradually.
Downing (2014)	--	1.30×	Increased global primary production of lakes and impoundments due to increased temperature alone. Reported is the median rate of increase for the latitudes with most ample agriculture and aquatic systems. Global water temperature is projected from known models of the relationship between ambient and surface water temperature and known relationships between aquatic primary production (directly related to P) and water temperature.
Downing (2014)	1.05×	1.10×	Change in the spatial extent of lakes and impoundments based on a conservative IPCC scenario of altered climate. This projection is an indicator of the magnitude of increased global run-off due to climate change and was derived from the data used by Tranvik et al. (2009), reanalyzed by Downing (2014) considering increased run off, melting of permafrost, and lost ice cover at high latitudes. The original publication reports 1.10× by 2100; here we estimated the change at 2050 by assuming a linear increase from present.

Supplementary Table 2. Global methane emissions from lakes and impoundments (95% confidence interval) under future scenarios where total phosphorus (TP) concentrations are 0.75, 1.5, 2, 2.5, and 3× (0.75×, 1.5×, 2×, 2.5×, 3×) that of current levels (1×). These simulated TP concentrations correspond to chlorophyll a (chla) concentrations that, on average, are 0.8, 1.3, 1.7, 2.0, and 2.2× (0.8×, 1.3×, 1.7×, 2.0×, 2.2×) that of current levels (1).

Scenario		Methane Emissions					
		Tg CH_4-C y^{-1}			*Pg C-CO_2-eq y^{-1}		
**TP**	**chla**	**Diffusive**	**Ebullitive**	**Total**	**Diffusive**	**Ebullitive**	**Total**
†0.75×	0.8×	24 (15-41)	57 (34-96)	95 (62-146)	0.30 (0.19-0.51)	0.7 (0.4-1.2)	1.2 (0.8-1.8)
1×	1×	28 (17-48)	67 (39-115)	112 (71-177)	0.34 (0.21-0.60)	0.8 (0.5-1.4)	1.4 (0.9-2.2)
1.5×	1.3×	33 (19-62)	84 (48-151)	141 (87-233)	0.41 (0.24-0.76)	1.0 (0.6-1.9)	1.7 (1.1-2.9)
2×	1.7×	38 (21-74)	99 (54-184)	167 (99-284)	0.46 (0.26-0.91)	1.2 (0.7-2.3)	2.1 (1.2-3.5)
2.5×	2.0×	42 (23-85)	112 (60-215)	189 (110-331)	0.51 (0.28-1.05)	1.4 (0.7-2.7)	2.3 (1.4-4.1)
3×	2.2×	45 (24-96)	124 (65-244)	210 (120-376)	0.56 (0.30-1.18)	1.5 (0.8-3.0)	2.6 (1.5-4.6)

*1 Tg CH_4 is equivalent to 34 Tg CO_2 on a 100-year time frame[4].

†The 0.75× was included to investigate the potential for reducing emissions through improved nutrient management.

Supplementary References

1. Mccauley E, Downing JA, Watson S. Sigmoid Relationships between Nutrients and Chlorophyll among Lakes. *Can J Fish Aquat Sci* **46**, 1171-1175 (1989).

2. Jeppesen E, *et al.* Climate change effects on nitrogen loading from cultivated catchments in Europe: implications for nitrogen retention, ecological state of lakes and adaptation. *Hydrobiologia* **663**, 1-21 (2011).

3. Cooke GD, Welch SA, Peterson SA, Nichols SA. *Restoration and Management of Lakes and Reservoirs.*, 3rd edn. Taylor & Francis (2005).

4. Downing JA. Marine nitrogen: Phosphorus stoichiometry and the global N:P cycle. *Biogeochemistry* **37**, 237-252 (1997).

5. Downing JA. Productivity of Freshwater Ecosystems and Climate Change. In: *Global Environmental Change* (ed^(eds Freedman B). Springer Netherlands (2014).

6. Caraco NF, Cole JJ. Human impact on nitrate export: An analysis using major world rivers. *Ambio* **28**, 167-170 (1999).

7. Cole JJ, Peierls BL, Caraco NF, Pace ML. Nitrogen loading of rivers as a human-driven process. In: *Humans as Components of Ecosystems* (ed^(eds McDonnell MJ, Pickett STA) (1993).

8. Samir KC, Lutz W. The human core of the shared socioeconomic pathways: Population scenarios by age, sex and level of education for all countries to 2100. *Global Environ Chang* **42**, 181-192 (2017).

9. Bouwman L, *et al.* Exploring global changes in nitrogen and phosphorus cycles in agriculture induced by livestock production over the 1900-2050 period. *Proceedings of the National Academy of Sciences of the United States of America* **110**, 20882-20887 (2013).

10. Tilman D, *et al.* Forecasting agriculturally driven global environmental change. *Science* **292**, 281-284 (2001).

11. Cordell D, Drangert JO, White S. The story of phosphorus: Global food security and food for thought. *Global Environ Chang* **19**, 292-305 (2009).

12. Jeppesen E, *et al.* Climate change effects on runoff, catchment phosphorus loading and lake ecological state, and potential adaptations. *J Environ Qual* **38**, 1930-1941 (2009).

13. Sinha E, Michalak AM, Balaji V. Eutrophication will increase during the 21st century as a result of precipitation changes. *Science* **357**, 405-408 (2017).
