# Supplementary material for: Eutrophication will increase methane emissions from lakes and impoundments during the 21st century
Source: Nat Commun. 2019 Mar 26;10:1375. doi: 10.1038/s41467-019-09100-5 (PMC6435651; doi:10.1038/s41467-019-09100-5)
Supplement: Supplementary file 3 — Description of Additional Supplementary Files [file 41467_2019_9100_MOESM3_ESM.pdf]

Description of Additional Supplementary Information

File Name: Supplementary Data 1

Description: Joint lake-size by chlorophyll a (chla) distributions for six TP loading scenarios (0.75x, 1x, 1.5x, 2x, 2.5x, or 3x).
